# O027. Sub-cortical sources of the somatosensory pathway are hypoactive in migraine interictally: a Functional Source Separation analysis

**DOI:** 10.1186/1129-2377-16-S1-A55

**Published:** 2015-09-28

**Authors:** Camillo Porcaro, Giorgio Di Lorenzo, Stefano Seri, Francesco Pierelli, Franca Tecchio, Gianluca Coppola

**Affiliations:** 1 LET'S-ISTC-CNR, Ospedale Fatebenefratelli, Isola Tiberina, Rome, Italy; 2 Laboratory of Psychophysiology, Psychiatric Clinic, Department of Neuroscience, University of Rome “Tor Vergata”, Rome, Italy; 3 The Wellcome Trust Laboratory for MEG Studies, School of Life and Health Sciences, Aston University, Birmingham, UK; 4 Sapienza University of Rome Polo Pontino, Latina, Italy; 5 G.B. Bietti Eye Foundation-IRCCS, Rome, Italy

## Background

Recent morpho-functional evidence pointed out that abnormalities in the thalamus could play a major role in the expression of migraine neurophysiological and clinical correlates. Whether this phenomenon is primary or secondary to its functional disconnection from the brainstem remains to be determined. We used a Functional Source Separation algorithm of EEG signal to extract the activity of the different neuronal pools recruited at different latencies along the somatosensory pathway in interictal migraine without aura (MO) patients.

## Methods

Twenty MO patients and 20 healthy volunteers (HV) underwent EEG recording. Four ad-hoc functional constraints, two sub-cortical (FS14 at brainstem and FS16 at thalamic level) and two cortical (FS20 radial and FS22 tangential parietal sources), were used to extract the activity of successive stages of somatosensory information processing in response to the separate left and right median nerve electric stimulation. A band-pass digital filter (450-750 Hz) was applied offline in order to extract high-frequency oscillatory (HFO) activity from the broadband EEG signal.

## Results

In both stimulated sides, significant reduced sub-cortical brainstem (FS14) and thalamic (FS16) HFO activations characterized MO patients when compared with HV. No difference emerged in the two cortical HFO activations between the two groups.

## Conclusions

Present results are the first neurophysiological evidence supporting the hypothesis that a functional disconnection of the thalamus from the subcortical monoaminergic system may underline the interictal cortical abnormal information processing in migraine. Further studies are needed to investigate the precise directional connectivity across the entire primary subcortical and cortical somatosensory pathway in interictal MO.

Written informed consent to publication was obtained from the patient(s).

